# Prevalence of and factors associated with depressive symptoms among the working population in Thimphu, Bhutan: A cross-sectional study

**DOI:** 10.1371/journal.pone.0347340

**Published:** 2026-04-21

**Authors:** Tshering Tshomo, Pilasinee Wongnuch, Peeradone Srichan

**Affiliations:** 1 Bhutan stroke foundation, Thimphu, Bhutan; 2 School of Health Science, Mae Fah Luang University, Chiang Rai, Thailand; University of Tabuk, SAUDI ARABIA

## Abstract

**Background:**

The working population contributes significantly to the economic and social development of a country. Despite an increase in mental health concerns in the working population, research on the mental health of Bhutan’s workforce is lacking.

**Objective:**

To evaluate the prevalence of depressive symptoms and identify associated factors in the working population in Thimphu, Bhutan.

**Method:**

A cross-sectional study was used to collect information from employees working in both the government and private sectors. The participants (aged 18–60 years) were selected using a stratified random sampling technique. A self-administered validated questionnaire was used to collect information. Depressive symptoms were assessed using the Patient Health Questionnaire (PHQ-9).

**Results:**

A total of 379 participants were recruited. The overall prevalence of depressive symptoms among the working population in Thimphu, Bhutan was 45.9%. Participants with primary school, high school, or a bachelor’s degree or higher education had significantly lower odds of depressive symptoms than those with no education. Likewise, those in supervisory and operational roles had lower odds than executives. Lower odds of depressive symptoms were also found among those without kidney disease, those with a family history of severe mental illness, and those whose job needs were understood to a limited or great extent. In contrast, moderate-to-severe anxiety and high levels of depersonalization were strongly associated with increased odds of depressive symptoms.

**Conclusion:**

Depressive symptoms were highly prevalent among formal-sector workers in Thimphu, Bhutan, indicating a substantial burden of mental health concerns within this specific urban workforce. The findings suggest the need for targeted workplace mental health interventions, particularly those that address supervisory support and individual vulnerabilities related to health and psychosocial stressors.

While this study contributes valuable insights, it reflects an urban, formal employment context, limiting its generalizability to Bhutan’s broader or informal labor force. These findings highlight the critical need for incorporating mental health promotion into workplace policies, particularly within urban employment settings. Moreover, a national mental health policy should formally recognize the role of occupational settings in promoting psychological well-being and allocating resources accordingly.

## Introduction

Depression is a common mental disorder that affects all age groups and is characterized by persistent sadness, emptiness, or irritability with cognitive and physical symptoms that impair daily life for at least two weeks [1]. According to the World Health Organization (WHO), an estimated 280 million people worldwide were living with depression as of 2025 [2]. Notably, an additional 53.2 million individuals were diagnosed with major depressive disorder following the COVID-19 pandemic [3]. The burden of depression is particularly high in Southeast Asia, (which includes Bhutan), where approximately 86 million individuals are currently affected [4,5]. Bhutan has a reported prevalence of depression of 4.0% [6], with a marked increase in incidence—from 9 cases per 10,000 individuals between 2011 and 2019–16 cases per 10,000 individuals in 2020 and further to 32 cases per 10,000 individuals in 2021 [7]. This upward trend has been identified as a key contributor to the increase in suicide attempts in the country. According to the Royal Bhutan Police Statistical Yearbook (2022), a total of 112 suicide cases were recorded in 2022, a significant number given Bhutan’s relatively small population of approximately 790,000 people [8]. Depression remains the most reported mental disorder among the general and working population [9].

According to the “Mental Health at Work” report, approximately 60% of the global population is employed [10], and depression remains a major mental health issue in the workplace, contributing to economic loss and reduced productivity [11]. Its prevalence varies across sectors—26% among health care professionals during the pandemic [12], 21% among industry workers [13], and 19% among teachers [14]. Depression affects workplace functioning through absenteeism, inconsistent performance, stigma, and strained work relationships [15]. The contributing factors include job strain, bullying [16], specific occupations, departmental roles, high demands [17], family conflict, low rewards, and poor social support [18]. Chronic diseases such as diabetes, heart disease, and kidney disease pose major challenges to global public health because of their long-term nature and incurability. For example, individuals with kidney disease are at increased risk of experiencing depressive symptoms, as are those with other chronic conditions [19]. Additionally, psychosocial risk factors—such as high job demands, limited control, inadequate support, and poor work conditions—have been linked to workplace depression [20]. In Bhutan, approximately 69.4% of the population is within the working age range (15–64 years) [21]. Workers are vital to national development and protected under the labor administration system.

Moreover, no research has been conducted among the working population in Bhutan to assess the prevalence of depression and its associated factors. In addition, there is no separate mental health policy or legislation in the country, and there is no proper implementation or linkage of mental health policy in the workplace or other sectors [22,23]. Furthermore, stigma and discrimination connected to mental health are universal concerns that are prominent in Bhutan, resulting in most people with mental disorders and depression receiving no treatment or delaying their seeking of help [24]. Additionally, the nation is experiencing a shortage of mental health practitioners. Currently, Bhutan has a total of 19 mental health specialists, including 3 psychiatrists serving the entire country who are stationed at Jigme Dorji Wangchuck National Referral Hospital in the capital city of Thimphu. In addition, published studies pertaining to mental health of the working population in Bhutan are scarce, as evidenced by the Mental Health Atlas of 2020 [23]. Thus, this study aimed to estimate the prevalence of depressive symptoms and to identify associated factors among the working population in Thimphu, Bhutan.

## Materials and methods

### Study design and setting

A cross-sectional study was conducted in Thimphu (the capital city of Bhutan). The population of the Thimphu district in 2021 was approximately 154,379; thus, it is the most densely populated region in the country. Moreover, people from all over the country reside in this district [21]. This district was selected because Thimphu District is considered to be among the most developed districts in the country and has a significant presence of both private and government sector organizations. According to a National Statistical Bureau report, approximately 54,853 people are employed in different sectors in the capital city of Thimphu, which contains the highest number of people in the country [21].

### Study population

The study participants included individuals who were employed, aged between 18 and 60 years, and who were actively working in both the government and private sectors within the Thimphu district. According to the International Labor Organization, the working-age population is defined as individuals aged 15–64 years. According to the labor and employment act of Bhutan, the minimum age of employment should be 18 years and above. In this study, employees aged 18–60 years were recruited.

### Sample size calculation and sampling technique

By using the sample size calculation formula for a cross-sectional study [25], at Z_α/2_ = 1.96, α = 0.05, and p = 0.442 [26], at least 379 samples were required for the analysis. Employees from the government and private sectors were selected using stratified random sampling, and eight different organizations were selected, with four originating from the government: the health, finance, education and engineering sectors. The private sector included the construction sector, tourism sector, hoteliers, and other offices.

### Conceptual framework

This study adopted elements from two well-established occupational health models: the Job Demands–Resources model [27] and the Effort–Reward Imbalance model [28]. These theoretical frameworks posit that psychological strain, including depression, arises when there is an imbalance between high job demands (e.g., workload, emotional stressors, and role ambiguity) and insufficient job resources (e.g., supervisory support, job control, and satisfaction). An imbalance between demand and resources increases vulnerability to adverse mental health outcomes. On the basis of these established models, this study selected variables representing both individual-level factors (e.g., education, position at work, anxiety, and kidney disease) and organizational-level factors (e.g., job satisfaction and perceived supervisor support). Psychosocial stressors such as having a family member with severe mental health problems were also included because of their potential impact on an individual’s emotional resilience and stress regulation.

### Research instruments

The questionnaire was administered in English, which is widely used and understood among the working population in Thimphu, particularly in government and private sector offices. The questionnaire was reviewed by three external experts: one medical doctor from the Ministry of Health in Bhutan and two academic experts from Mae Fah Luang University, Thailand. The item-objective congruence (IOC) method was applied to evaluate each item’s alignment with the study objectives. Items scoring below 0.5 were removed, those scoring between 0.5 and 0.7 were revised according to expert feedback, and items scoring above 0.7 were retained. A pilot test was subsequently conducted with a sample of 30 individuals from the target population to assess both the clarity and reliability of the questionnaire. The pilot results indicated a high level of internal consistency, with a Cronbach’s alpha coefficient of 0.878, supporting the tool’s reliability in the Bhutanese context.

The sociodemographic characteristics, behavioral factors, physical health information, and work-related factors of the participants were collected using a validated self-administered questionnaire. Job burnout was assessed using the Maslach Burnout Inventory (MBI) under license from Mind Garden, Inc. Emotional exhaustion (EE) was classified as low (0–16), moderate (17–26), and high (≥27); depersonalization (DP) was classified as low (0–6), moderate (7–12), and high (≥13); and personal accomplishment (PA) was classified as low (≥39), moderate (32–38), and high (0–31).

The Generalized Anxiety Scale (GAD-7) was utilized to assess anxiety. This 7-item questionnaire assesses anxiety levels over the past two weeks using a four-point Likert scale (0–3). Total scores range from 0 to 21, with higher scores indicating more severe anxiety: 0–4 (none to minimal), 5–9 (mild), 10–14 (moderate), and 15–21 (severe). This scale has shown excellent internal consistency (Cronbach’s α = 0.92) [29]; in this study, Cronbach’s α was 0.874. Depressive symptoms were assessed using the PHQ-9, a 9-item scale aligned with DSM-IV criteria. Scores were interpreted as none (0–4), mild (5–9), moderate (10–14), moderately severe (15–19), and severe (20–27). This scale has shown excellent internal reliability (Cronbach’s α = 0.892) [30]; this study reported a Cronbach’s α of 0.881. The questionnaire comprises four sections. The first section contains 12 items related to sociodemographic characteristics and assesses age, sex, education, religion, marital status, number of children, family size, family conflicts, and debt. The second section contains 8 items related to behavioral factors and assesses alcohol consumption, tobacco use, and substance use. The third section contains 7 items on physical health and assesses COVID-19 status, kidney disease, hypertension, diabetes, family history of chronic or mental illness, and disability. The fourth section contains 14 items on work-related factors, and assesses employment sector, job type, position, salary, work experience, weekly working hours, department size, job satisfaction, supervisor support, and relationships in the workplace.

### Data collection procedures

Ethical approval was obtained from the Mae Fah Luang University Ethics Committee. Organizational heads were contacted and informed about the study objectives. Data collection was conducted from August to September 2023. Participants were selected voluntarily and provided with detailed information regarding the study’s purpose, procedures, benefits, and potential risks. Written informed consent was obtained. Participants were assured of their right to withdraw at any time. Confidentiality was maintained using coded forms without personal identifiers.

### Ethical considerations

The present study was approved by the Mae Fah Luang University Ethics Committee on Human Research (EC 23068−18). All the participants were asked to provide written informed consent. All the respondents’ answers were kept confidential. The forms contained only identification numbers and did not include any personal information that could identify individual participants.

### Statistical analysis

Descriptive statistics are presented as frequencies and percentages. Associations were assessed using chi-square or Fisher’s exact tests. All the statistical tests were two-tailed with a significance level of α = 0.05. No formal correction for multiple comparisons was applied, as all analyses were based on a priori hypotheses. However, the risk of Type I error due to multiple testing is acknowledged. Depressive symptoms were categorized as none-to-mild (No) and moderate-to-severe (Yes). Multiple logistic regression models were used to identify associated factors, with odds ratios (ORs), adjusted odds ratios (AORs) and 95% confidence intervals (CIs) reported.

Variables for the multivariable analysis were selected on the basis of a conceptual framework. Additionally, variables with p < 0.20 in the univariable analyses were considered for inclusion. To assess potential overfitting, we conducted diagnostic evaluations, including the Hosmer–Lemeshow goodness-of-fit test and multicollinearity checks using variance inflation factors (VIFs). All the VIF values were less than 2.0, indicating that there was no evidence of multicollinearity. The final model demonstrated acceptable fit and stability.

## Results

A total of 379 participants were included, with the majority being male (55.9%) and a mean age of 32.29 years (range: 19–57); most were under 40 years. More than half of the participants (55.1%) had completed high school, and 31.4% held a bachelor’s degree or higher. The majority were Buddhist (92.9%), followed by Hindu (5.3%) and Christian (1.8%). Most of the participants were married (69.4%), while 23.5% were single, 6.9% were divorced, and 0.3% were widowed. With respect to household size, 64.4% lived with three or more family members, 25.9% lived with two, and 9.8% lived alone. Approximately 30.1% reported partner conflict, and 15.6% reported debt, mostly under Nu 50,000 (mean = Nu 160,461.02). Alcohol consumption was low, with 42.7% never drinking and 43.8% drinking monthly or less; 69.4% never consumed hard liquor, and 66.8% avoided local brews. Hypertension and diabetes were reported by 16.4% and 7.1% of the participants, respectively, with some unsure of their status. Kidney disease was reported by 6.3% of the participants, while 9.0% had a family member with chronic illness and 5.3% had a family member with severe mental illness (S1 Table).

Half of the participants worked in the government sector (50.1%). The largest occupational group was hoteliers (20.1%). Most held operational roles (53.0%), with 26.1% in supervisory/support roles, 14.8% in professional/management, and 6.1% in executive/specialist roles. Regular employment was reported by 63.6% of participants, contract employment by 34.3%, and temporary employment by 2.1%. The most common monthly income was Nu 12,000–20,000, which was reported by 38.3% of the participants. Most (57.0%) had worked >20 years, with a mean duration of 19.34 years. The number of working hours per week ranged from 50–69 hours (68.1%) to 30–49 hours (13.7%). Most departments had < 30 workers (81.5%), with a mean of 15.99. Approximately 49.3% were satisfied or extremely satisfied with their jobs. Most participants (74.7%) rated supervisor understanding as “to a limited extent”. Furthermore, 57.5% of participants reporting receiving help/support from supervisors “sometimes”, whereas 33.0% and 9.5% reporting receiving help/support “always” or “never”, respectively. Additionally, 50.9% reported that their supervisors were “sometimes” willing to listen, while 38.8% reported this as “always,” and 10.3% as “never.” Supervisor relationships were rated as good (59.9%), fair (36.7%), and poor (3.4%). Coworker relationships were similarly rated as good (59.6%), fair (36.2%), and poor (4.2%) (Table 1).

**Table 1:** Work-related factors of the participants.

Characteristics	n	%
**Working Sector**		
Government sector	190	50.1
Private sector	189	49.9
**Occupational Sector**		
Education sector (Teachers)	72	19.0
Engineer	28	7.4
Finance sector	43	11.3
Health sector (Nurses/doctors)	47	12.4
Construction sectors	62	16.3
Tourism sector	42	11.1
Hoteliers	76	20.1
Others (call center)	9	2.4
**Position at work**		
Executives and Specialist	23	6.1
Professional and management	56	14.8
Supervisory and support	99	26.1
Operational	201	53.0
**Employment type**		
Regular	241	63.6
Contract	130	34.3
Others (temporary)	8	2.1
**Monthly salary (Ngultrum)**		
Less than Nu.12000	59	15.6
Nu.12000-Nu.20000	145	38.3
Nu.20000-Nu.30000	81	21.3
More than Nu.30000	94	24.8
**Duration in the current job (years)**		
1-10	75	19.8
11-20	88	23.2
>20	216	57.0
Mean = 19.34, SD = 9.27, Min = 1, Max = 31		
**Working hours per week**		
Less than 30hrs/week	26	6.9
30-49hrs/week	52	13.7
50-69hrs/week	258	68.1
≥70 hrs/week	43	11.3
**Total numbers of workers in the department**		
1-29	309	81.5
30-59	67	17.7
>60	3	0.8
Mean = 15.99, SD = 15.76, Min = 1, Max = 150		
**Satisfaction with the current job**		
Very dissatisfied	26	6.9
Dissatisfied	18	4.7
Neutral	148	39.1
Satisfied to Extremely satisfied	187	49.3
**Supervisor understands jobs problem and needs**		
Not at all	25	6.6
To a limited extent	283	74.7
To a high extent	71	18.7
**Supervisors help and support**		
Never	36	9.5
Sometimes	218	57.5
Always	125	33.0
**Supervisor willingness to listen to work-related problems**		
Never	39	10.3
Sometimes	193	50.9
Always	147	38.8
**Relation with supervisor**		
Poor	13	3.4
Fair	139	36.7
Good	227	59.9
**Relation with co-worker**		
Poor	16	4.2
Fair	137	36.2
Good	226	59.6

As shown in Table 2, 55.4% of the participants reported low levels of emotional exhaustion, 31.9% reported moderate levels, and 12.7% reported high levels. With respect to depersonalization, 44.3% reported low levels, 34.0% reported moderate levels, and 21.7% reported high levels. In terms of personal accomplishment, 89.7% scored high, 5.0% scored moderate, and 5.3% scored low. With respect to anxiety levels, 29.8% of the participants experienced minimal anxiety, 37.5% experienced mild anxiety, 26.6% experienced moderate anxiety, and 6.1% experienced severe anxiety. With respect to depressive symptoms, 54.1% of participants were classified as “No” (none-to-mild symptoms), whereas 45.9% were classified as “Yes” (moderate-to-severe symptoms).

**Table 2:** Prevalence of Job Burnout, Anxiety, and depressive symptoms among the working population in Thimphu, Bhutan.

Characteristics	n	%
**The Maslach Burnout Inventory (MBI) Scale**		
**Emotional Exhaustion (EE)**		
Low	210	55.4
Moderate	121	31.9
High	48	12.7
**Depersonalization (DP)**		
Low	168	44.3
Moderate	129	34.0
High	82	21.7
**Reduced Personal Accomplishment (PA)**		
Low	20	5.3
Moderate	19	5.0
High	340	89.7
**Level of Anxiety**		
Minimal anxiety	113	29.8
Mild anxiety	142	37.5
Moderate anxiety	101	26.6
Severe anxiety	23	6.1
**Level of Depressive symptoms**		
No (none-to-mild)	205	54.1
Yes (moderate-to-severe)	174	45.9

In the multivariable logistic regression analysis, depressive symptoms were dichotomized into none-to-mild (54.1%) and moderate-to-severe (45.9%). Eight variables were significantly associated with depressive symptoms. Educational attainment was a protective factor: participants with a primary school education (AOR = 0.035; 95% CI: 0.001–0.844; p = 0.039), high school education (AOR = 0.019; 95% CI: 0.001–0.404; p = 0.011), or a bachelor’s degree or higher (AOR = 0.006; 95% CI: 0.001–0.152; p = 0.002) had significantly lower odds of depressive symptoms than those with no education. Employment in supervisory (AOR = 0.118; 95% CI: 0.024–0.577; p = 0.008) and operational (AOR = 0.167; 95% CI: 0.037–0.749; p = 0.019) roles was also associated with lower odds of having depressive symptoms than executive roles. Participants without kidney disease (AOR = 0.171; 95% CI: 0.053–0.549; p = 0.003) and those with a family history of severe mental illness (AOR = 0.164; 95% CI: 0.039–0.692; p = 0.014) were less likely to experience depressive symptoms. Moderate-to-severe anxiety was strongly associated with depressive symptoms (AOR = 23.283; 95% CI: 10.996–49.301; p < 0.001). Perceived supervisor understanding of job problems had a protective effect, both to a limited extent (AOR = 0.138; 95% CI: 0.030–0.635; p = 0.011) and to a great extent (AOR = 0.088; 95% CI: 0.016–0.477; p = 0.005). Higher levels of depersonalization were significantly associated with increased odds of depressive symptoms, with both moderate (AOR = 4.095; 95% CI: 2.038–8.229; p < 0.001) and high (AOR = 7.252; 95% CI: 3.119–16.864; p < 0.001) levels showing this association. Finally, in the univariable analysis, participants who were very dissatisfied with their current job had significantly higher odds of having depressive symptoms (OR = 5.263, 95% CI: 1.903–14.552; p = 0.001) than those who were satisfied/extremely satisfied (Table 3).

**Table 3:** Univariable and multivariable analyses to identify the factors associated with depressive symptoms among the working population of Thimphu, Bhutan.

Factors	Depressive symptoms		Univariable Analysis			Multivariable Analysis		
	Yes n(%)	No n(%)	OR	95%CI	p-value	AOR	95%CI	p-value
**Education**								
No education	9(81.8)	2(18.2)	1.00			1.00		
Primary school	19(47.5)	21(52.5)	0.201	0.038-1.050	0.057	0.035	0.001-0.844	0.039*
High school	107(51.2)	102(48.8)	0.233	0.049-1.105	0.067	0.019	0.001-0.404	0.011*
Bachelor’s degree and higher	39(32.8)	80(67.2)	0.108	0.022-0.526	0.006*	0.006	0.001-0.152	0.002*
**Position at work**								
Executives and Specialist	14(60.9)	9(39.1)	1.00			1.00		
Professional and management	28(50.0)	28(50.0)	0.643	0.239-1.726	0.381	0.361	0.079-1.654	0.190
Supervisory and support	47(47.5)	52(52.5)	0.581	0.230-1.466	0.250	0.118	0.024-0.577	0.008*
Operational	85(42.3)	116(57.7)	0.471	0.195-1.139	0.095	0.167	0.037-0.749	0.019*
**Kidney disease**								
Yes	15(62.5)	9(37.5)	1.00			1.00		
Don’t know	23(69.7)	10(30.3)	1.380	0.454-4.191	0.570	0.538	0.108-2.666	0.448
No	136(42.2)	186(57.8)	0.439	0.186-1.032	0.059	0.171	0.053-0.549	0.003*
**Severe mental health problems among family members**								
Yes	7(35.0)	13(65.0)	0.659	0.256-1.693	0.386	0.164	0.039-0.692	0.014*
Don’t know	19(63.3)	11(36.7)	2.112	0.974-4.579	0.058	0.832	0.190-3.648	0.807
No	148(45.0)	181(55.0)	1.00			1.00		
**Level of Anxiety**								
Minimal to Mild	64(25.1)	191(74.9)	1.00			1.00		
Moderate to Severe	110(88.7)	14(11.3)	23.449	12.563-43.767		23.283	10.996-49.301	<0.001*
**Supervisor understands jobs problem and needs**								
Not at all	16(64.0)	9(36.0)	1.00			1.00		
To a limited extent	133(47.0)	150(53.0)	0.499	0.213-1.166	0.108	0.138	0.030-0.635	0.011*
To a great extent	25(35.2)	46(64.8)	0.306	0.118-0.791	0.015*	0.088	0.016-0.477	0.005*
**Depersonalization (DP)**								
Low	39(23.3)	129(76.8)	1.00			1.00		
Moderate	74(57.4)	55(42.6)	4.450	2.699-7.338	<0.001*	4.095	2.038-8.229	<0.001*
High	61(74.4)	21(25.6)	9.608	5.212-17.713	<0.001*	7.252	3.119-16.864	<0.001*
**Satisfaction with the current job**								
Very dissatisfied	21(80.8)	5(19.2)	5.263	1.903-14.552	0.001*	4.089	0.990-16.885	0.052
Dissatisfied	6(33.3)	12(66.7)	0.627	0.226-1.740	0.370	0.008	0.001-0.084	<0.001*
Neutral	64(43.2)	84(56.8)	0.955	0.618-1.475	0.834	0.902	0.461-1.763	0.762
Satisfied/Extremely satisfied	83(44.4)	104(55.6)	1.00			1.00		

## Discussion

The overall prevalence of depressive symptoms among the working population in Thimphu, Bhutan, was 45.9%, with participants experiencing moderate to severe levels of depressive symptoms. Eight factors were associated with depressive symptoms: education, position at work, kidney disease status, severe mental health problems among family members, anxiety, supervisor understanding of job-related problems and needs, job burnout (depersonalization), and job satisfaction. These findings highlight the multifaceted nature of mental health challenges in the workplace and underscore the importance of addressing both personal health and organizational factors to improve the psychological well-being of workers.

The prevalence of depressive symptoms in our study was greater than that reported by studies among workers in the Republic of North Macedonia, Skopje (11.0%) [31], Nepal (15.0%) [32], Vietnam (38.6%) [33] and Bangladesh (23.5%) [34]. However, another study in Bangladesh reported a markedly higher prevalence of depression (61.5%) than that reported in our findings [35]. The variations in the presence of depressive symptoms reported in these studies can be explained by differences in working structures, facilities, policies, sample sizes, and working sectors.

This study revealed that higher education was significantly associated with lower odds of depressive symptoms. Participants with primary, high school, or bachelor’s degrees and above were less likely to report depressive symptoms than those with no formal education. This aligns with longitudinal evidence from Indonesia showing that higher educational attainment predicts reduced depression risk over time [36] and with findings from the U.S. indicating that higher education is linked to lower odds of depressive symptoms [36]. Higher education may promote mental well-being through improved job prospects, financial security, and stronger social networks.

Employment position was significantly associated with depressive symptoms. Participants in supervisory/support and operational roles had notably lower odds of depressive symptoms than those in executive and specialist roles. These results suggest that individuals in more routine or manual roles may experience less psychological distress than those in higher-responsibility or decision-making roles. One possible explanation is that executives and specialists may face greater work-related stress, including long hours, management responsibilities, and performance pressure, which could increase vulnerability to depression, as supported by systematic reviews [37]. A study conducted in the United States reported that executives often experience higher levels of work-related stress, primarily because of the pressure to meet high expectations for productivity and performance [38]. High job demands coupled with limited control create a high-strain environment that exacerbates mental health risks. These findings highlight the need to address mental health stressors specific to leadership roles in workplace interventions. Leadership-focused mental health interventions should prioritize communication, interpersonal interaction, and leadership style, as these factors significantly influence the well-being of individuals in leadership roles [39]. In the Bhutanese context, tailored interventions that address executive-specific stressors are most effective when organizational strategies (e.g., workload adjustments and role clarity) are combined with individual approaches (e.g., resilience training and stress management). These integrated efforts support leaders’ mental well-being and contribute to healthier, more supportive workplaces across both the public and private sectors.

In our study, we found an association between kidney disease and depression. Psychiatric disorders usually cooccur with a wide range of chronic illnesses, particularly chronic kidney disease (CKD) [19]. Another study reported that the prevalence of depression among patients with CKD was 24.6% [40]. Additionally, the prevalence of depression among people with chronic illnesses in Bhutan was reported to be 41.0% [41]. A study conducted among chronic disease patients reported that the prevalence of moderate depression was 45.1%, that of moderate-to-severe depression was 12.4%, and that of severe depression was 4.6% [40].

Interestingly, compared with those without such a history, participants who reported a family history of severe mental health problems had significantly lower odds of experiencing depressive symptoms. This unexpected association may reflect greater mental health awareness, earlier recognition of symptoms, or proactive help-seeking behaviors among individuals with familial exposure to mental illness. Families with prior experience caring for a member with mental illness are often better equipped to adapt and respond to challenging situations. Educational support provided to such families regarding mental health conditions and available treatments can help reduce depressive symptoms and enhance their capacity to provide effective care [42]. A qualitative study revealed that family members with prior experience navigating the mental health care system, despite the challenges involved, played a vital role in caregiving. Their accumulated experience enhanced their adaptability and responsiveness in managing complex situations related to their relative’s mental illness [43]. Previous studies suggest that increased mental health literacy and perceived vulnerability can lead to increased engagement with preventive or therapeutic measures, potentially mitigating the onset or severity of depression [44]. Alternatively, this finding may be an artifact arising from statistical instability due to sparse data in some exposure categories, as suggested by wide confidence intervals in the multivariable model. Reporting bias—such as underreporting mental illness in families due to stigma—or residual confounding by unmeasured psychosocial or protective factors (e.g., family support and coping resources) may also explain this unexpected direction of association. These issues underscore the need to interpret this finding with caution and suggest that replication in larger or more balanced samples is warranted.

We also found a strong association between depression and anxiety among workers. According to a study conducted among individuals residing in the Community of Selangor, Malaysia, the prevalence of comorbid depression and anxiety was 67.2% [45]. According to the results of their study, individuals with anxiety had a 12.82-fold greater likelihood of experiencing depression (95% CI = 9.88–16.63). Anxiety and other negative emotional states, particularly depression, are significantly correlated with one another [46] and are highly comorbid [47].

We found that employees with leaders who do not understand their needs and job problems are more likely to experience depression. Leaders are the core of any enterprise, and their leadership behavior significantly influences employees [48]. The leader’s behavior and the extent to which a leader cultivates an environment that supports employees are important factors in reducing mental health symptoms. Moreover, positive leadership qualities within an organization are related to fewer mental health symptoms and greater employee well-being [49].

In this study, participants who reported being very dissatisfied with their job satisfaction were significantly more likely to experience depressive symptoms compared with those who were satisfied or extremely satisfied. This finding is consistent with evidence from other settings. Similarly, a cross-sectional study in Peru revealed that job dissatisfaction was significantly associated with increased odds of depressive symptoms [50]. Similarly, a study among Japanese civil servants aged 19–65 years reported that compared with satisfied employees, those who were dissatisfied with their jobs had a 1.94 times greater risk of depression [49]. A meta-analysis involving 43,884 rural-to-urban migrant workers in China also demonstrated a strong association between low job satisfaction and depressive symptoms [51]. Furthermore, research among Chinese adults aged 35–60 years confirmed an inverse relationship between job satisfaction and depression, indicating that dissatisfaction is linked to increased emotional distress, reduced motivation, and poor work performance [52]. These findings underscore the importance of promoting job satisfaction to support mental well-being and workplace productivity. However, in the current study, the association between job satisfaction and depressive symptoms was observed only in the univariable analysis and was not maintained in the multivariable model after adjustment for potential confounders. This discrepancy suggests that the unadjusted association may be influenced by confounding factors. Therefore, the observed association between job satisfaction and depressive symptoms warrants careful interpretation because of potential confounding and model instability. To clarify this association, future research should employ longitudinal study designs that can better capture temporal and causal relationships.

In terms of policy and institutional implications, the lack of a dedicated national mental health policy or legislation in Bhutan limits the capacity for systematic responses to mental health issues. This gap constrains the development and implementation of mental health policy initiatives such as employee counseling services, workplace-based mental health training, the promotion of psychologically safe work environments, and organizational mechanisms that support employee well-being. With respect to institutional capacity constraints, the country faces a critical shortage of trained mental health professionals, including psychiatrists, clinical psychologists and psychiatric nurses. Moreover, mental health training is often insufficiently integrated into health care providers, resulting in limited competencies at the primary care level. National budget allocations should explicitly support the training, recruitment, and retention of a qualified mental health workforce as a critical step toward strengthening service delivery and addressing the growing mental health burden. In the workplace structure system, labor policies should include mental health standards and monitoring mechanisms, such as mental health screening protocols and legal protection against workplace stigma and discrimination. Furthermore, occupational health strategies should incorporate structured interventions—such as mental health literacy training for managers and stress management workshops. Mental health interventions should be integrated into the education sector to promote early-life development of self-management skills and facilitate timely recognition of psychological distress. Integrating mental health literacy into school and university programs can provide individuals with the knowledge and capacity to identify early warning signs of mental health conditions that enhance help-seeking behavior and support smoother pathways in the health care system.

This study has a few limitations. One key limitation of this study involves the issue of statistical uncertainty, particularly in relation to the sparse data and wide confidence intervals observed in some model estimates. These wide intervals suggest a lack of precision in the estimated effect sizes, potentially due to small cell counts or unbalanced distributions of responses across predictor categories. This is a common concern in observational studies where subgroup sizes may be small, leading to unstable or inflated estimates in maximum likelihood-based models. Therefore, the results related to key predictors such as anxiety and severe mental health problems among family members should be interpreted with caution. Despite statistically significant or notable effect sizes, the presence of sparse data and wide confidence intervals in these variables may reflect model instability or estimation bias rather than precise associations. This study was conducted within the working population of Thimphu, the capital city of Bhutan. However, it is important to note that the findings are applicable solely within this specific setting. Furthermore, the sample of occupational categories chosen for this study is limited, perhaps resulting in a lack of representativeness for the broader working population inside the country.

## Conclusions

There is a high prevalence of depressive symptoms among formal-sector workers in Thimphu, Bhutan, although the findings are limited to urban formal employment settings. Several factors were significantly associated with depressive symptoms, including educational level, job position, the presence of kidney disease, anxiety symptoms, severe mental health problems in the family, a lack of supervisor support, job burnout (particularly depersonalization), and low job satisfaction. These findings underscore the complex interplay between individual health, workplace dynamics, and mental well-being. Importantly, higher education may play a protective role through pathways such as enhanced employment opportunities, financial stability, and expanded social capital. The presence of supportive workplace environments—particularly where supervisors recognize and respond to employees’ psychosocial needs—appears vital to mental health outcomes. Furthermore, addressing job dissatisfaction and burnout is essential not only for individual well-being but also for organizational performance and sustainability. Targeted workplace interventions—at both the individual and organization levels—are urgently needed to reduce the burden of depressive symptoms. Such interventions should integrate mental health promotion into occupational health policies and encourage supportive leadership practices. These insights are particularly relevant for Bhutan, where work-related mental health remains underprioritized but increasingly critical.

## Supporting information

S1 Table General characteristics of the participants. (DOCX)

## Abbreviations

WHO: World Health Organization
MBI: maslach burnout inventory
EE: emotional exhaustion
DP: depersonalization
PA: personal accomplishment
GAD-7: the generalized anxiety scale
OR: odds ratios
AOR: adjusted odds ratios
CI: confidence intervals
CKD: chronic kidney disease
VIF: variance inflation factors
