# Supplementary material for: Prevalence of and factors associated with depressive symptoms among the working population in Thimphu, Bhutan: A cross-sectional study
Source: PLoS One. 2026 Apr 21;21(4):e0347340. doi: 10.1371/journal.pone.0347340 (PMC13098953; doi:10.1371/journal.pone.0347340)
Supplement: S1 Table — (DOCX) [file pone.0347340.s001.docx]

**S1 Table. General characteristics of the participants.**

| **Characteristics** | **n** | **%** |
| --- | --- | --- |
| **Total** | 379 | 100.0 |
| **Sex** |  |  |
| Male | 212 | 55.9 |
| Female | 167 | 44.1 |
| **Age** (years) |  |  |
| <30 | 166 | 43.8 |
| 31-40 | 166 | 43.8 |
| ≥41 | 47 | 12.4 |
| Mean = 32.29, Minimum = 19, Maximum = 57, SD = 6.55 | | |
| **Education level** |  |  |
| No education | 11 | 2.9 |
| Primary school | 40 | 10.6 |
| High school | 209 | 55.1 |
| Bachelor’s degree and higher | 119 | 31.4 |
| **Religion** |  |  |
| Buddhism | 352 | 92.9 |
| Hinduism | 20 | 5.3 |
| Christianity | 7 | 1.8 |
| **Marital Status** |  |  |
| Single | 89 | 23.5 |
| Married | 263 | 69.4 |
| Divorced | 26 | 6.9 |
| Widowed | 1 | 0.3 |
| **Total number of family members** |  |  |
| 1 | 37 | 9.8 |
| 2 | 98 | 25.9 |
| 3 and more | 244 | 64.4 |
| **Conflicts with the partner** |  |  |
| Yes | 114 | 30.1 |
| No | 265 | 69.9 |
| **Debt** |  |  |
| Yes | 59 | 15.6 |
| No | 320 | 84.4 |
| **Total debt owned** |  |  |
| <Nu 50000 | 338 | 89.2 |
| Nu 50000-150000 | 26 | 6.9 |
| >Nu 150000 | 15 | 4.0 |
| Mean = 160,461.02, Min = 10,000, Max = 800,000, SD = 186,882.65 | | |
| **Consume drinks containing alcohol** |  |  |
| Never | 162 | 42.7 |
| Monthly or less | 166 | 43.8 |
| 2 to 4 times a month | 41 | 10.8 |
| 2 to 3 times a week | 3 | 0.8 |
| 4 or more times a week | 7 | 1.8 |
| **Beer consumption** |  |  |
| Never | 197 | 52.0 |
| Monthly or less | 144 | 38.0 |
| 2 to 4 times a month | 30 | 7.9 |
| 2 to 3 times a week | 2 | 0.5 |
| 4 or more times a week | 6 | 1.6 |
| **Consume hard drinks like Whisky, vodka, etc.** |  |  |
| Never | 263 | 69.4 |
| Monthly or less | 82 | 21.6 |
| 2 to 4 times a month | 29 | 7.7 |
| 2 to 3 times a week | 2 | 0.5 |
| 4 or more times a week | 3 | 0.8 |
| **Consume local drinks like Ara, bangchang and changkoe** |  |  |
| Never | 253 | 66.8 |
| Monthly or less | 94 | 24.8 |
| 2 to 4 times a month | 24 | 6.3 |
| 2 to 3 times a week | 3 | 0.8 |
| 4 or more times a week | 5 | 1.3 |
| **Hypertension** |  |  |
| Yes | 62 | 16.4 |
| Don’t know | 28 | 7.4 |
| No | 289 | 76.3 |
| **Diabetes** |  |  |
| Yes | 27 | 7.1 |
| Don’t know | 29 | 7.7 |
| No | 323 | 85.2 |
| **Kidney disease** |  |  |
| Yes | 24 | 6.3 |
| Don’t know | 33 | 8.7 |
| No | 322 | 85.0 |
| **Chronic disease among family members** |  |  |
| Yes | 34 | 9.0 |
| Don’t know | 25 | 6.6 |
| No | 320 | 84.4 |
| **Severe mental health problems among family members** |  |  |
| Yes | 20 | 5.3 |
| Don’t know | 30 | 7.9 |
| No | 329 | 86.8 |
